# Effects of rhythmic auditory stimulation on motor function and balance ability in stroke: A systematic review and meta-analysis of clinical randomized controlled studies

**DOI:** 10.3389/fnins.2022.1043575

**Published:** 2022-11-17

**Authors:** Lei Wang, Jin-lin Peng, Wu Xiang, Yi-jie Huang, Ai-lian Chen

**Affiliations:** ^1^Department of Rehabilitation Medicine, Hunan Provincial People's Hospital, The First Affiliated Hospital of Hunan Normal University, Changsha, China; ^2^Tongji Medical College, Tongji Hospital, Huazhong University of Science and Technology, Wuhan, China; ^3^Department of Rehabilitation, Beibei Traditional Chinese Medical Hospital, Chongqing, China; ^4^Department of Rehabilitation Medicine, The Seventh Affiliated Hospital, Sun Yat-sen University, Guangzhou, China

**Keywords:** stroke, motor disorders, rehabilitation, rhythmic auditory stimulation, motor fluctuations

## Abstract

**Objective:**

Rhythmic auditory stimulation (RAS) belongs to neurologic music therapy, which has attracted clinical attention because of its efficacy in motor function after stroke. This study aimed to summarize the effectiveness of rhythmic auditory stimulation (RAS) for the treatment of motor function and balance ability in stroke through a systematic review and meta-analysis.

**Methods:**

All studies were retrieved from six databases. The effects of RAS on stroke were determined using the following indicators: motor function including step length, step cadence, velocity, Fugl–Meyer assessment (FMA); and balance ability including overall balance index (OBI) and Berg Balance Scale (BBS). The risk map of bias of the quality of the studies and the meta-analysis results of the indicators was prepared using RevMan 5.2 software.

**Results:**

A total of 1,363 abstracts were retrieved. Among them, 325 duplicate studies were eliminated, and 971 studies were excluded after reading the titles and abstracts. In addition, by downloading the full text for further reading and screening, 47 studies were excluded. A total of 22 studies were included in the systematic review, and 18 studies were included in the meta-analysis. Assessment of quality, based on the PEDro scale, two studies had low quality, three studies had excellent quality, and the other studies had good quality; based on the Cochrane Collaborative Network Bias Risk Assessment Scale. A total of 15 studies specifically explained the random methods used. Meanwhile, seven studies did not report random sequence generation. A total of 10 studies reported that the evaluation of experimental results was blinded. In the meta-analysis, the results of motor function [namely, velocity (SMD = 0.99, 95% CI (0.43, 1.55)), step length (SMD = 0.97, 95% CI (0.74, 1.20)), and step cadence (MD = 5.16, 95% CI (4.17, 6.14)), FMA (MD = 2.93, 95% CI (2.04, 3.83))], were statistically significant (*P* < 0.01). The results of balance ability [OBI (MD = −0.51, 95% CI (−0.86, −0.16)) and BBS (MD = 2.93, 95% CI (1.67, 4.20))], were also statistically significant (*P* < 0.01). Among all the outcome indicators, three indicators were included in more than 10 studies: these are step length, step cadence, and velocity. The results showed that the two sides of the funnel chart were asymmetrical, thus these results all showed heterogeneity. The GRADEpro GDT online tool was used to evaluate the quality of evidence for the outcome indicators in the included studies. Five outcome indicators were included, of which three were low-quality indicators and two were moderate-quality indicators.

**Conclusions:**

RAS could improve gait parameters, walking function, and balance ability of individuals with stroke. However, studies or samples of outcome indicators for balance ability of stroke patients is relatively insufficient, which also requires further research in the future.

**Systematic review registration:**

PROSPERO, identifier: CRD42021225102.

## Introduction

Stroke is the second leading cause of death and the third leading cause of disability worldwide (Feigin et al., 2022), with more than 13 million new stroke cases annually (GBD 2016 Neurology Collaborators, 2019). In the 2022 World Stroke Organization (WSO) report, from 1990 to 2019, stroke events and stroke-related deaths increased by 70.0 and 43.0%, respectively (Feigin et al., 2022). Recently, stroke mortality has declined with improved medical care and preventive measures, however, the absolute number of new stroke cases annually has increased, thereby leading to a growing burden of stroke-related disability (Feigin et al., 2014; Platz, 2019). One-third of patients with stroke are permanently disabled, and more than half of stroke survivors aged 65 years and older have mobility impairment (Virani et al., 2020). Thus, these trends will continue with the development of the population on average as people are growing older (Platz, 2019).

Motor dysfunction is one of the most common consequences of stroke and includes impairment of coordination and postural control (Langhorne et al., 2009). About 60% of stroke patients have difficulty walking (Mehrholz et al., 2014) because of motor and sensory disturbances on the hemiplegic side, in addition to symptoms, such as spasticity and cognitive impairment that may further hinder walking. Therefore, gait recovery is often the focus of rehabilitation efforts to enhance not only physical activity but also autonomy and participation in daily life (Mainka et al., 2018). Therefore, gait recovery is the most important goal of rehabilitation programs for patients with stroke (Lee et al., 2018). In clinical practice, some therapeutic methods are used to treat motor dysfunction after stroke and show good efficacy, such as Neurologic music therapy, repetitive transcranial magnetic stimulation(rTMS) (Fan et al., 2021) and Virtual reality (Turolla et al., 2013).

Neurologic music therapy, a crucial complementary therapy, is currently used in the rehabilitation of movement, speech, and cognition and is accepted in the medical field (Thaut et al., 2014). Rhythmic auditory stimulation (RAS) is a technology in neurologic music therapy and is based on rhythmic and repetitive auditory stimuli (Yoo and Kim, 2016). RAS uses an external rhythm (music) to facilitate internally generated rhythmic movements, such as walking (Thaut, 2015). In addition, RAS can be used as auditory cues for walking and may facilitate internal neural timing among post-stroke patients from a neurophysiological perspective (Ghai and Ghai, 2019). One of the earliest and relevant studies about the use of RAS in stroke rehabilitation was conducted by Thaut et al. (1993): they observed that RAS can effectively reduce stride time variability and more balanced muscle activation patterns between hemiplegic and unaffected limbs. Moreover, RAS may lead to improved lower extremity and gait function after stroke and can easily be used as adjunctive therapy (Thaut, 2015). Suh et al. also confirmed that RAS with significant effects on improving balance as well as gait coordination and symmetry (Suh et al., 2014), Yang et al. also confirmed this conclusion (Yang et al., 2016). Furthermore, several studies support the use of gait training with RAS in the chronic phase of stroke because it improves walking speed and flexibility (Ko et al., 2016; Wright et al., 2017). In the last 20 years, the of RAS for stroke treatment has been widely investigated. Nascimento et al. (2015), which provided evidence that rhythm cue training can improve walking speed and stride length more than walking training alone. It can also benefit from the rhythm and symmetry of walking. This review includes only seven trials with a total of 211 participants, including randomized controlled trials (RCT) and clinical controlled trials (CCT). Yoo and Kim (2016) in a review obtained the beneficial effects of RAS through meta-analysis, which confirmed that RAS can improve the gait parameters and other motor functions of patients, thus supporting its application in widening the rehabilitation field of stroke patients. The study was published in 2016, and only 10 studies (RCT or CCT) were included in the meta-analysis, with a total of 356 subjects. However, the above two studies have some limitations in the number of articles, the number of participants, and the quality of studies (Nascimento et al., 2015; Yoo and Kim, 2016). In addition, those studies' systematic reviews did not involve research on the balance ability of stroke patients. Therefore, the scientific basis of the influence of RAS on gait should be based on high-quality evidence-based medicine by incorporating more high-quality literature.

Thus, this study aimed to determine the effect of rhythmic auditory stimulation on the rehabilitation of patients with stroke and movement disorders through a systematic review and meta-analysis of the literature. This study will include more RCT studies to further improve the quantity and quality of articles to improve the evidence of evidence-based medicine. This work also provides strong evidence for the use of RAS to treat stroke and analyze the deficiencies of previous studies.

## Methods

Herein, a systematic review was planned and conducted based on the Preferred Reporting Items for Systematic Reviews and Meta-Analyses (PRISMA) statement (Cumpston et al., 2019) and was registered with PROSPERO (registration number CRD42021225102). A PRISMA checklist is provided in Supplementary material 1.

### Search strategy

Two reviewers (Lei Wang and Jin-lin Peng) performed electronic searches in the following publication databases in July 2022 without restrictions on publication year: Medline, Web of Science, Embase, PubMed, Wanfang, and China National Knowledge Infrastructure (CNKI). Various combinations of keywords were used as search terms, including stroke, cerebrovascular disorders, intracranial infarction, intracranial hemorrhage, hemiplegia; music, music therapy, rhythmic cueing, and rhythmic auditory stimulation. Moreover, the key terms matched the appropriate Medical Subject Headings (MeSH) terms. Pre-searches were performed, and the final search style was selected as follows: PUBMED: ((((((((music[Title/Abstract]) OR (music therapy[Title/Abstract])) OR (rhythmic cueing[Title/Abstract])) OR (rhythmic auditory stimulation[Title/Abstract])) OR (music[MeSH Terms])) OR (music therapy[MeSH Terms])) OR (rhythmic cueing[MeSH Terms])) OR (rhythmic auditory stimulation[MeSH Terms])) AND ((((((((((stroke[MeSH Terms]) OR (cerebrovascular disorders[MeSH Terms])) OR (intracranial infarction[MeSH Terms])) OR (intracranial hemorrhage[MeSH Terms])) OR (hemiplegia[MeSH Terms])) OR (hemiplegia[Title/Abstract])) OR (intracranial hemorrhage[Title/Abstract])) OR (intracranial infarction[Title/Abstract])) OR (cerebrovascular disorders[Title/Abstract])) OR (stroke[Title/Abstract])). Meanwhile, a manual search (an online search of relevant journals and references of review articles) was conducted to identify papers that may have been missed in the electronic database search.

### Eligibility criteria

#### Inclusion criteria

The Population, Intervention, Comparison, Outcomes, Study Design (PICOS) framework (Cristini et al., 2021) was used to establish the eligibility criteria for the articles to be included in the review. For the population, studies on participants diagnosed with stroke were included. For intervention, studies that used RAS as intervention and had a well-defined protocol that included information on the specific training parameters (type, time, intensity, frequency, and duration) were included. For comparison, studies should include interventions in the control group (such as drugs, rehabilitation training, etc.). For outcomes (for meta-analysis), studies that evaluated motor function including step length, cadence, and velocity; Fugl–Meyer assessment (FMA), balance ability including overall balance index (OBI), and the Berg Balance Scale (BBS) were included. Study Design: Only RCTs were included in the review.

#### Exclusion criteria

Studies involving animal research, conference research, protocol study, or computer model research as well as duplicate papers were excluded. Two reviewers (Lei Wang and Jin-lin Peng) independently screened the titles and abstracts to identify articles that meet the inclusion criteria. The full-text versions of the identified articles were obtained and separately screened to ensure that they met the inclusion criteria. Meanwhile, a third reviewer (Ai-lian Chen) made the final assessment about whether or not full-text papers met the inclusion criteria. The whole screening process should follow the PICOS framework.

### Study selection

Two authors (LW and J-lP) independently reviewed the title and abstract sections of the retrieved articles. First, duplicate studies were eliminated by using the “Medical Literature King V6” software (Beijing Yimai Communication Technology Co., Ltd., Beijing, China). Second, studies were ruled out that did not meet the inclusion criteria based on the title and abstract of the remaining articles after duplicate checking under the guidance of the PICOS framework in the eligibility criteria. Finally, potentially relevant studies were downloaded for a more detailed full-text review. If the results of the two independent authors differ, then a third author (Ai-lian Chen) will participate in the discussion and decide the final consensus.

### Data extraction

Herein, the following data were extracted: general information including first author, sample size, gender, age, treatment course, and intervention measures; outcome indicators: gait parameters including step length, cadence, and velocity; motor function including FMA, OBI, and BBS. Two authors (Lei Wang and Wu Xiang) independently reviewed the data based on the search strategy. If the results of the two independent authors differ, then a third author (Ai-lian Chen) will participate in the discussion and decide the final consensus. When an included article had no valid data, the author of the article will be contacted. However, if data were still unavailable, then the article was not included in the meta-analysis but was included in the systematic review.

### Quality and risk of bias assessment

Quality evaluation of the included studies was performed independently by two reviewers (Wu Xiang and Lei Wang) and was revised by a third reviewer (Jin-lin Peng). The methodological quality of the intervention studies was assessed using the Physiotherapy Evidence Database (PEDro) scale. The PEDro scale is a valid and reliable measure of the methodological quality of RCTs. This 10-item scale is based on core criteria for RCT quality assessment (Elbanna et al., 2019). Based on the PEDro scale, the quality of papers was classified as follows: studies with scores of lower than six points were considered low-quality studies, whereas studies with scores equal to or >6 points were considered high-quality studies (6–7 is good and 8–10 is excellent quality) (Maher et al., 2003).

GRADEpro GDT online tool was used to evaluate the quality of the evidence of outcome indicators including five degrading factors: risk of bias, inconsistency, indirectness, imprecision, and other considerations. The quality of evidence can be divided into 4 levels: “high,” “moderate,” “low,” and “very low.”

Then, we evaluated the quality of the included studies. Scores were compared in a consensus meeting by two independent authors (Wang Lei and Peng Jin-lin). If the results of the two independent authors differ, then a third author (Yi-jie Huang) will participate in the discussion and decide the final consensus. In addition, the Cochrane risk of bias assessment tool was used to assess the risk of bias in the articles. Each article was assessed for selection bias, performance bias, detection bias, attrition bias, and reporting bias. Each domain was rated as high risk of bias, unclear of bias, or low risk of bias (Choi et al., 2020). Moreover, a risk map of the bias of the studies was prepared with RevMan 5.2 software (Copenhagen: The Nordic Cochrane Center, The Cochrane Collaboration, 2014).

### Statistical analysis

Separate meta-analyses were conducted considering the heterogeneity of the interventions and measures of outcome indicators. Sub-group meta-analyses and sensitivity analyses were used to determine whether or not the characteristics of the interventions have any influence on the effects of RAS on PD. Meanwhile, the Review Manager 5.2 software of Cochrane Collaboration was used in the meta-analysis. The outcome variables were continuous, hence, the mean difference (MD) was calculated. The 95% CI of the statistical results was reported. A *P* < 0.05 indicated statistical significance for an overall effect (Z). Meanwhile, the Chi-square test was used to calculate the heterogeneity of the included articles. When heterogeneity was *P* > 0.1 and *I*^2^ < 50%, a fixed-effect model was used; when heterogeneity was *I*^2^ > 50%, the causes of heterogeneity were analyzed by subgroup or sensitivity analysis. When the results still had heterogeneity, the random-effect mode was used for summary analysis (Choi et al., 2020).

## Results

### Search results

**Figure 1:**
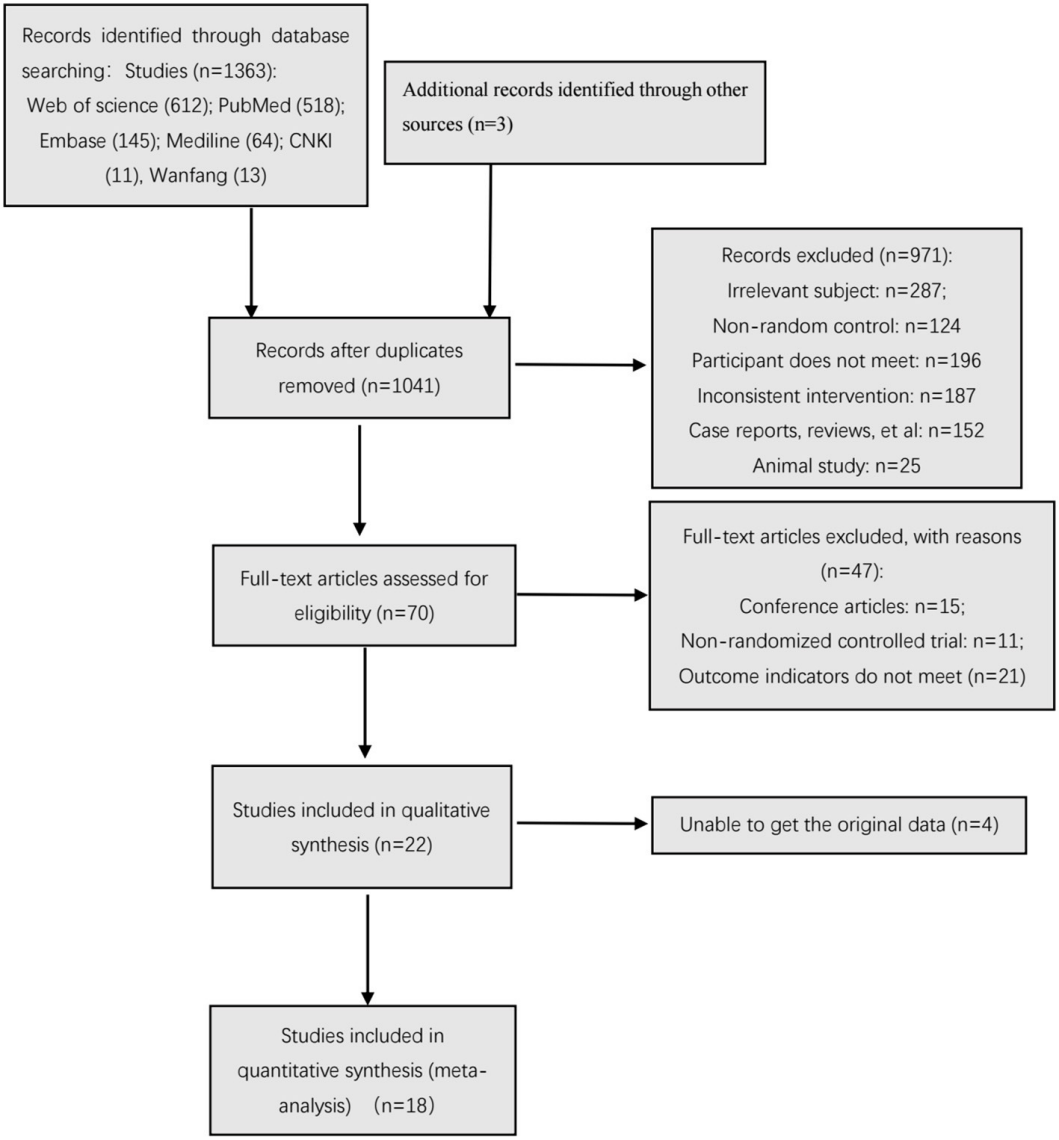
Flow chart of the search process.

In the different stages of retrieval and screening, different numbers of studies were also excluded. The detailed reasons and procedures are shown in Figure 1. A total of 1,363 abstracts were retrieved and imported into the Document Management Software of “Medical Literature King V6.” Among them, 325 duplicate studies were eliminated, and 971 studies were excluded after reading the titles and abstracts. In addition, 47 studies were excluded by downloading the full text for further reading and screening. Meanwhile, a total of 22 studies were included in the qualitative analysis. After the article outcome indicators were read, the original data of four studies were not obtained even after contacting the studies' authors; as such, these studies were excluded. Finally, 18 studies were included in the meta-analysis.

### Quality and risk of bias assessment

The studies evaluated based on the PEDro scale are listed in Table 1. Two studies (Kim et al., 2012; Yu-ge et al., 2016) had low quality, three studies (Thaut et al., 2007; Mainka et al., 2018; Elsner et al., 2020) had excellent quality, and the other studies had good quality.

**Table 1:** Quality assessment (PEDro scale) of included studies.

**Pedro scale questions**	**Q1**	**Q2**	**Q3**	**Q4**	**Q5**	**Q6**	**Q7**	**Q8**	**Q9**	**Q10**	**Q11**	**Total score**
Li-chun et al. (2011)	Y	Y	N	Y	N	N	N	Y	Y	Y	Y	6
Da-ao et al. (2014)	Y	Y	N	Y	N	N	N	Y	Y	Y	Y	6
Yu-ge et al. (2016)	Y	Y	N	Y	N	N	Y	N	N	Y	Y	5
Juan et al. (2019)	Y	Y	N	Y	N	N	N	Y	Y	Y	Y	6
Qiang et al. (2021)	Y	Y	N	Y	N	N	Y	Y	Y	Y	Y	7
Elsner et al. (2020)	Y	Y	Y	Y	N	N	Y	Y	Y	Y	Y	8
Mainka et al. (2018)	Y	Y	Y	Y	Y	Y	Y	N	N	Y	Y	8
Thaut et al. (1997)	Y	Y	N	Y	N	N	Y	Y	Y	Y	Y	7
Thaut et al. (2007)	Y	Y	Y	Y	H	H	Y	Y	Y	Y	Y	8
Schauer and Mauritz (2003)	Y	Y	N	Y	N	N	Y	Y	Y	Y	Y	7
Cha et al. (2014)	Y	Y	Y	Y	N	N	N	Y	Y	Y	Y	7
Kim and Oh (2012)	Y	Y	Y	Y	N	N	N	Y	Y	Y	Y	7
Lee et al. (2018)	Y	Y	N	Y	N	N	Y	Y	N	Y	Y	6
Kim et al. (2012)	Y	Y	N	Y	N	N	N	Y	N	Y	Y	5
Yoon and Kang (2016)	Y	Y	N	Y	N	N	N	Y	Y	Y	Y	6
Bunketorp-Kall et al. (2019)	Y	Y	Y	Y	N	N	Y	Y	N	Y	Y	7
Song and Ryu (2016)	Y	Y	N	Y	N	N	N	Y	Y	Y	Y	6
Yang et al. (2016)	Y	Y	N	Y	N	N	Y	Y	N	Y	Y	6
Cho and Kim (2020)	Y	Y	N	Y	N	N	Y	Y	Y	Y	Y	7
Suh et al. (2014)	Y	Y	Y	Y	N	N	N	Y	Y	Y	Y	7
Wang et al. (2021)	Y	Y	N	Y	N	N	N	Y	Y	Y	Y	6
Park et al. (2010)	Y	Y	N	Y	N	N	N	Y	Y	Y	Y	6

**Figure 2:**
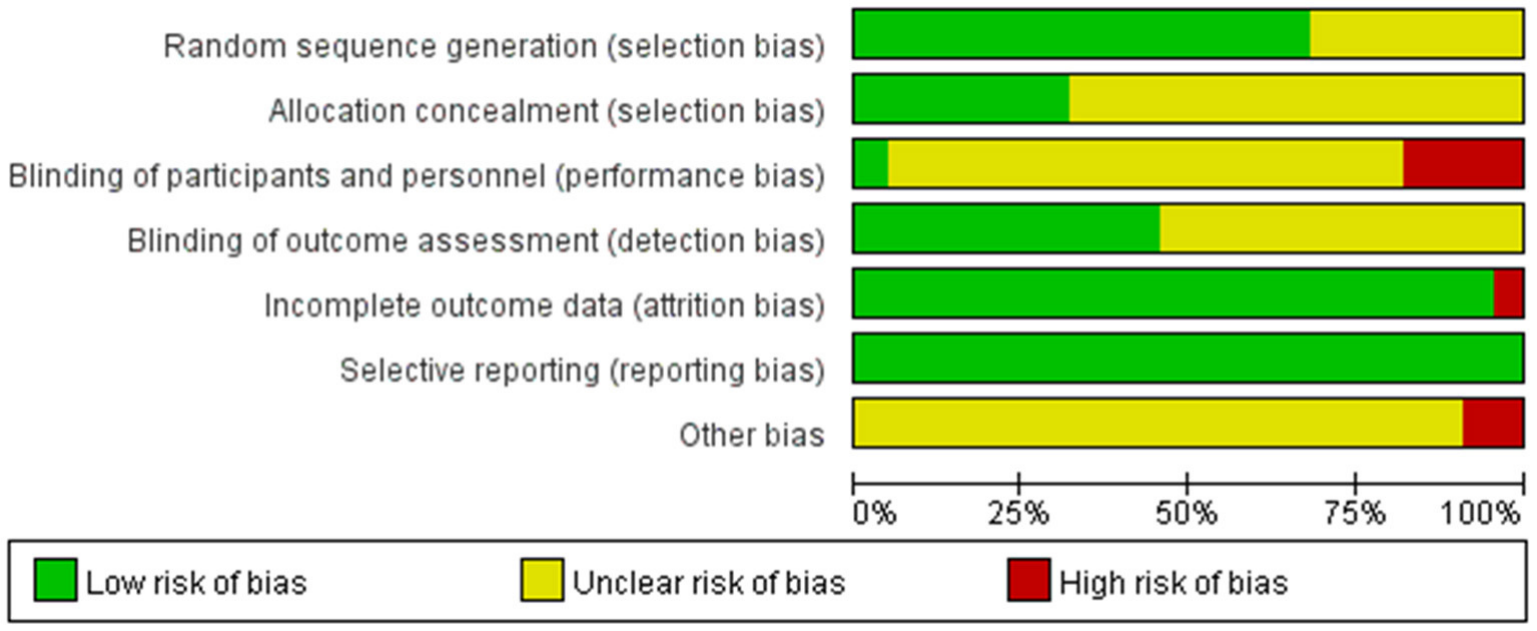
Risk of bias graph.

**Figure 3:**
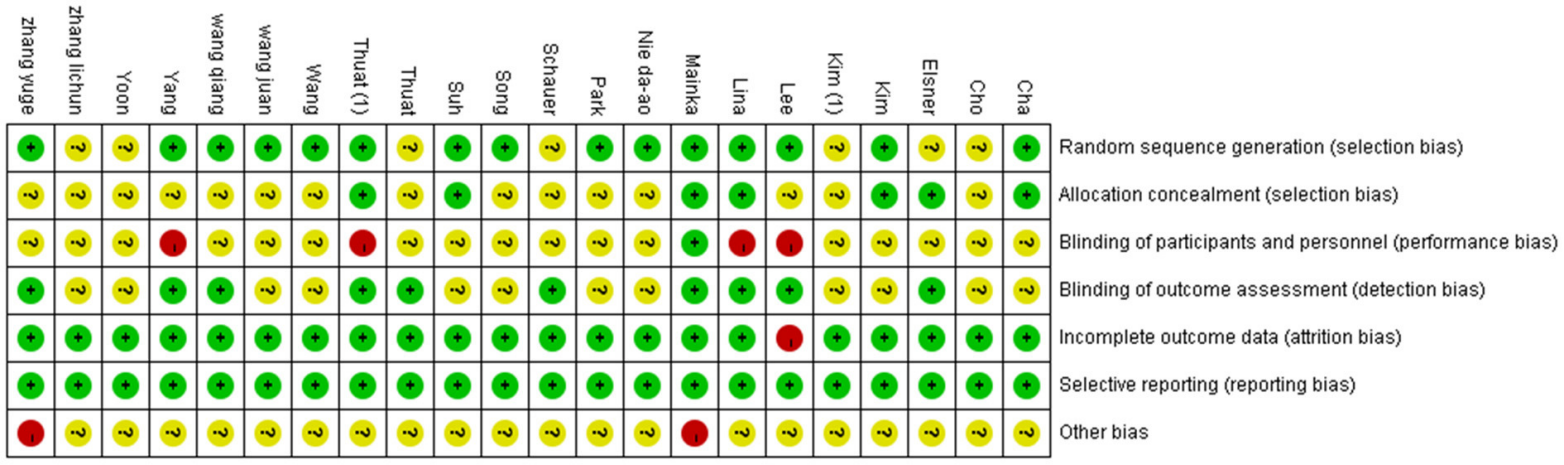
Risk of bias summary.

The results of the evaluation using the Cochrane Collaborative Network Bias Risk Assessment Scale are shown in Figures 2, 3. A total of 15 studies specifically explained the random methods used. However, seven studies did not report random sequence generation. Seven studies described allocation concealment. Three studies blinded the participants and persons involved. Moreover, 10 studies reported that the evaluation of experimental results was blinded. Only one study had a high-risk bias, whereas the other studies that reported bias and attrition bias had a low risk of bias.

### Study characteristics

The included studies had the following information: first author, sample size, age, gender, and diagnosis criteria. As shown in Table 2, the data included in the studies were as follows: the content of the intervention program, duration of RAS, number of RAS sessions, outcomes measured, and assessment time points. Among the included studies, the lowest sample size are 11 (Yu-ge et al., 2016), and the highest are 82 (Bunketorp-Kall et al., 2019). The intervention methods in the control group were various, included Traditional rehabilitation intervention, neuro developmental treatment, action observation therapy and treadmill training, et al. Intervention time varies greatly, ranging from 3 to 12 weeks. As shown in Tables 2, 3.

**Table 2:** Characteristics of participants in the included studies.

**Study**	**NIHSS**	**Course**	**Age (year)**	**Gender (M/ F)**	**Sample (*n*)**
	**C/E**	**C/E**	**C/E**	**C/E**	**C/E**
Li-chun et al. (2011)		8.00 ± 2.55 7.70 ± 2.71 8.75 ± 2.22 8.36 ± 1.57	67.00 ± 2.55 66.30 ± 4.38 67.25 ± 0.96 68.64 ± 2.62	5/10 4/11	15/15
Da-ao et al. (2014)	13.3 ± 2.6 13.4 ± 2.9	45 (16 + 149) 60 (23 + 143)	59 ± 16 61 ± 14	11/8 12/8	20/20
Yu-ge et al. (2016)	4.60 ± 1.95 5.33 ± 2.16	82.40 ± 46.92 77.67 ± 49.49	48.00 ± 17.46 43.33 ± 7.87	4/1 6/0	5/6
Juan et al. (2019)		91.30 ± 27.96 88.35 ± 31.13	57.15 ± 10.17 55.80 ± 9.84	12/8 11/9	20/20
Qiang et al. (2021)		76.53 ± 11.59 74.09 ± 10.16	62.32 ± 9.15 63.47 ± 8.69	17/17 19/15	34/34
Elsner et al. (2020)		99.2 ± 88.5 34.7 ± 20.1	65.3 ± 7.5 68.7 ± 11	2/4 1/5	6/6
Mainka et al. (2018)		46.9 ± 23.3 42.6 ± 30.1	65.5 ± 8.5 63.7 ± 8.8	2/11 4/7	11/13
Thaut et al. (1997)		15.7 ± 4 16.1 ± 4	72 ± 8 73 ± 7	5/5 5/5	10/10
Thaut et al. (2007)		22.2 ± 12 21.3 ± 11	69.7 ± 11 69.2 ± 11	19/16 22/21	35/43
Schauer and Mauritz (2003)			61 ± 12 59 ± 12		12/11
Cha et al. (2014)	26.1 ± 1.8 26.6 ± 2.1	14.7 ± 5.4 14.5 ± 5.5	63.0 ± 14.1 59.8 ± 11.7	6/4 6/4	10/10
Kim and Oh (2012)		15.3 ± 3.0 15.8 ± 2.3	64.5 ± 8.1 65.2 ± 6.8		10/10
Lee et al. (2018)		14.29 ± 5.16 14.22 ± 5.79	54.92 ± 6.65 56.00 ± 9.39	11/10 13/10	21/23
Kim et al. (2012)	27.4 ± 2.63 26.5 ± 2.59		51.8 ± 13.7 58.3 ± 11.8	7/3 6/4	9/9
Yoon and Kang (2016)		13.6 ± 8.5 16.4 ± 10.3	56.3 ± 7.1 50.8 ± 14.4	6/3 6/4	9/10
Bunketorp-Kall et al. (2019)	2.8 ± 3.6 3.0 ± 2.9		63.7 ± 6.7 62.7 ± 6.7	22/19 23/18	41/41
Song and Ryu (2016)	25.18 ± 0.8 25.30 ± 1.2	14.75 ± 6.0 12.30 ± 3.4	60.10 ± 6.8 57.10 ± 7.8	9/11 12/8	20/20
Yang et al. (2016)	27.45 ± 1.86 27.64 ± 1.56	11.97 ± 3.53 11.18 ± 3.68	55.82 ± 13.58 51.91 ± 13.30	9/2 9/2	11/11
Cho and Kim (2020)	26.93 ± 1.62 27 ± 1.41	26.13 ± 6.58 30.33 ± 7.69	59.07 ± 5.62 55.67 ± 7.62	9/6 11/4	15/15
Suh et al. (2014)	22.38 ± 7.73 24.50 ± 4.90	224.25 ± 213.03 386.38 ± 283.22	70.63 ± 12.42 61.00 ± 14.48	3/5 3/5	8/8
Wang et al. (2021)		8.45 ± 2.11 8.39 ± 2.09	61.02 ± 7.51 61.12 ± 7.49	10/20 8/22	30/30
Park et al. (2010)		14.00 ± 8.00 15.50 ± 5.00	52.90 ± 13 59.20 ± 11	8/4 8/5	12/13

C, control group; E, experiment group; M, male; F, female.

**Table 3:** Characteristics of study design in the included studies.

**Study**	**Interventions (C)**	**Interventions (E)**	**Assessment time points**	**Intensity minutes, frequency, duration**	**Outcome measures**
Li-chun et al. (2011)	T	C + RAS	After 5 months of treatment	Walk for 10 min, rest for 2 min, repeat 3 times, 2 times/d, 5 wks	Velocity, Cadence, Step length
Da-ao et al. (2014)	T	C + RAS	After 30 days of treatment	Walk for 10 min, rest for 3 min, repeat 3 times, 1 time/d, 30 days	FMA, velocity, Cadence, Step length
Yu-ge et al. (2016)	T	C + RAS	After 3 weeks of treatment	15 min, 5 times/week, 3 weeks	Velocity, Cadence, Step length
Juan et al. (2019)	T	C + RAS	After 4 weeks of treatment	20 min, 5 times/week, 4 weeks	FMA, BBS, velocity
Qiang et al. (2021)	T + AOT	C + RAS	After 8 weeks of treatment	15 min, repeat 2 times, 1 time/d, 8 weeks	FMA, BBS
Elsner et al. (2020)	Overground gait exercises	C + RAS	After 4 weeks of treatment	30 min, 3 times/week, 4 weeks	Velocity, Cadence, Step length, BBS
Mainka et al. (2018)	NDT + TT	C + RAS	After 4 weeks of treatment	15 min, 5 times/week, 4 weeks	Velocity, Cadence, Step length
Thaut et al. (1997)	NDT	C + RAS	After 6 weeks of treatment	30 min, 5 times/week, 6 weeks twice	Velocity, Cadence, Step length
Thaut et al. (2007)	NDT	C + RAS	After 3 weeks of treatment	30 min, 5 times/week, 3 weeks	Velocity, Cadence, Step length
Schauer and Mauritz (2003)	NDT	C + RAS	After 3 weeks of treatment	20 min, 5 times/week, 3 weeks	Velocity, Cadence, Step length
Cha et al. (2014)	T + underwent intensive gait training	C + RAS	After 6 weeks of treatment	30 min, 5 times/week, 6 weeks	Velocity, Cadence, Step length, BBS
Kim and Oh (2012)	Walked comfortably and safely over the ground	C + RAS	After 6 weeks of treatment	10 min, 3 times/week, 6 weeks	Velocity, Step length
Lee et al. (2018)	Gait training + T	C + RAS	After 6 weeks of treatment	30 min, 5 times/week, 6 weeks	FMA, BBS, TUG, velocity, Cadence
Kim et al. (2012)	T	C + RAS	After 5 weeks of treatment	30 min, 3 times/week, 5 weeks	TUG, velocity, Cadence
Yoon and Kang (2016)	inclined treadmill	C + RAS	After 4 weeks of treatment	30 min, 5 times/week, 4 weeks	TUG, BBS, Cadence, velocity
Bunketorp-Kall et al. (2019)	T	C + RAS	After 12 weeks of treatment	30 min, 2 times/week, 12 weeks	
Song and Ryu (2016)	NDT + gait training	C + RAS	After 4 weeks of treatment	30 min, 5 times/week, 4 weeks	Cadence, step length
Yang et al. (2016)	T + Treadmill training	C + RAS	After 4 weeks of treatment	30 min, 3 times/week, 4 weeks	Velocity, cadence, Step length
Cho and Kim (2020)	AOT and T	C + RAS	After 8 weeks of treatment	30 min, 3 times/week, 8 weeks	OBI
Suh et al. (2014)	Gait training+ NDT.	C + RAS	After 4 weeks of treatment	15 min, 5 times/week, 4 weeks	Velocity, length, cadence
Wang et al. (2021)	Conventional drug therapy, rehabilitation training, and walking training	C + RAS	After 4 weeks of treatment	60 min, 6 times/week, 4 weeks	FMA, BBS, TUG, Cadence, step length
Park et al. (2010)	Walking training with NDT	C + RAS	After 2 weeks of treatment	30 min, twice a day, 5 days a week, 2 weeks	Velocity

T, Traditional rehabilitation intervention; C, control group; E, experiment group; RAS, rhythmic auditory stimulation; NDT, neuro developmental treatment; AOT, action observation therapy; TT, treadmill training; FMA, Fugl-Meyer assessment; TUG, timed up and go test; BBS, Berg balance scale; OBI, overall balance index.

### Outcomes

#### Motor function

**Figure 4:**
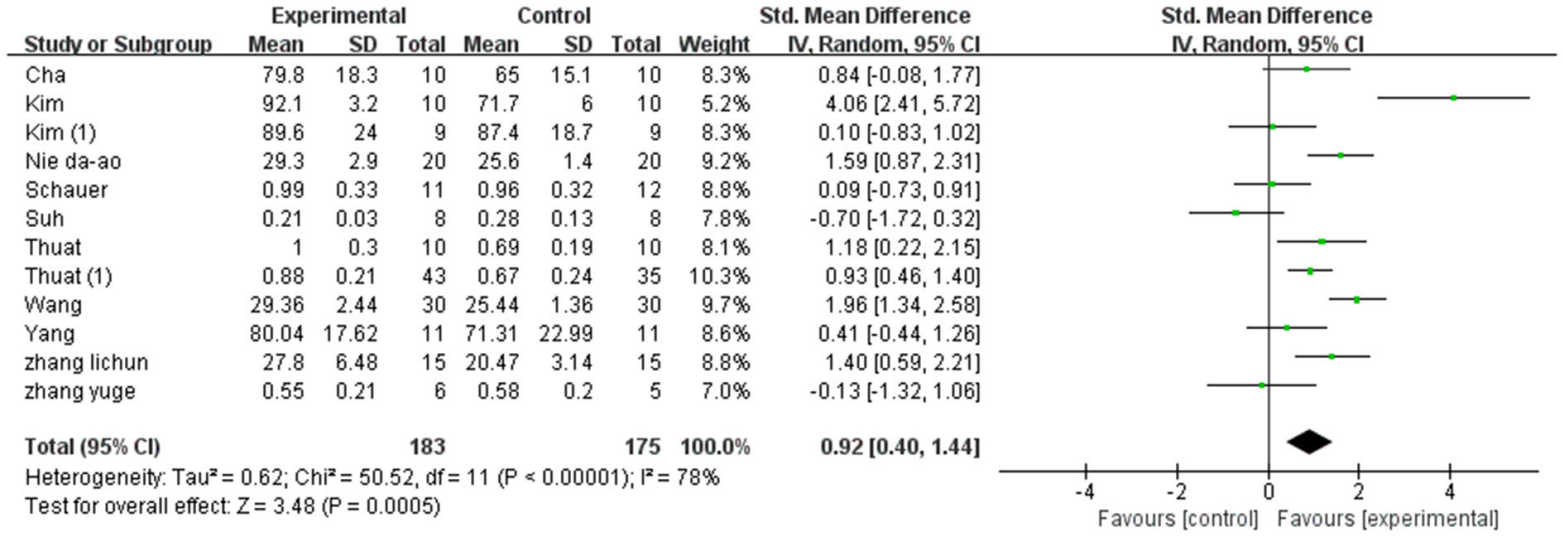
Forest plot of step length for meta-analysis.

A total of 358 participants were included in 12 studies on step length. Herein, the results showed heterogeneity (*I*^2^ = 78%). However, the subgroup and sensitivity analyses showed no significant change in heterogeneity. Therefore, we selected the random-effect model [SMD = 0.97, 95% CI (0.74, 1.20), *P* < 0.01]. Herein, the difference between the two groups was statistically significant (Figure 4).

**Figure 5:**
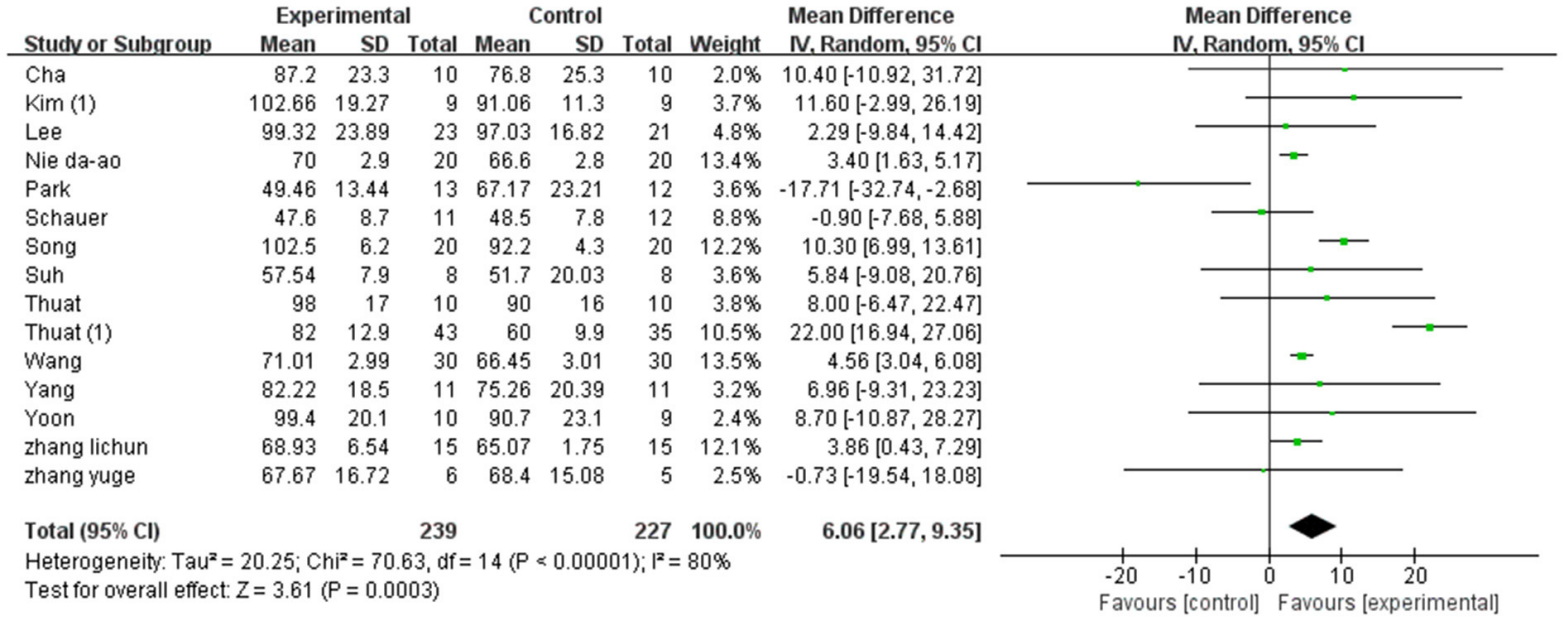
Forest plot of step cadence for meta-analysis.

A total of 466 participants were included in 15 studies on step cadence. Herein, the results showed heterogeneity (*I*^2^ = 80%). However, the result of the subgroup and sensitivity analyses showed no significant change in heterogeneity. Therefore, we selected the random-effect model [MD = 5.16, 95% CI (4.17, 6.14), *P* < 0.01]. Herein, the difference between the two groups was statistically significant (Figure 5).

**Figure 6:**
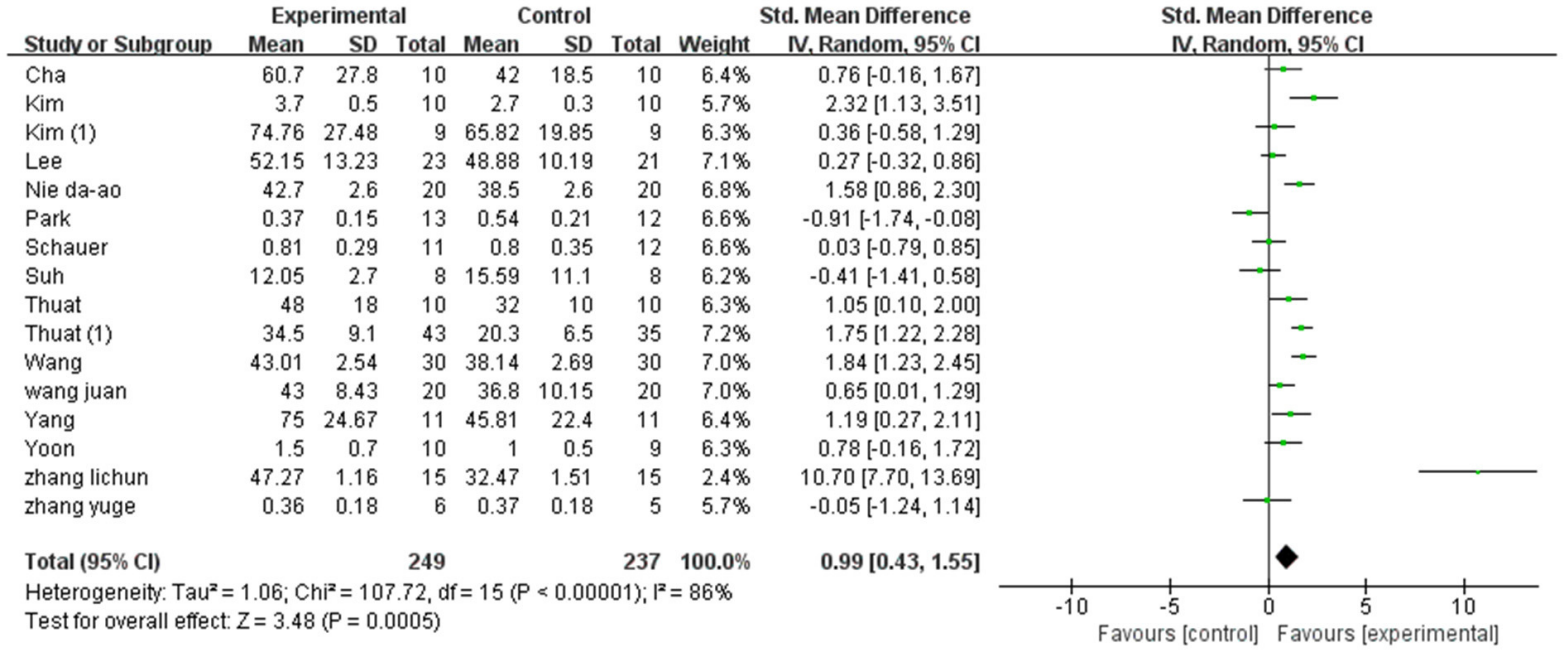
Forest plot of velocity for meta-analysis.

A total of 486 participants were included in 16 studies on velocity. Herein, the results showed heterogeneity (*I*^2^ = 86%). However, the result of the subgroup and sensitivity analyses showed no significant change in heterogeneity. Therefore, we selected the random-effect model [SMD = 0.99, 95% CI (0.43, 1.55), *P* < 0.01]. Herein, the difference between the two groups was statistically significant (Figure 6).

**Figure 7:**
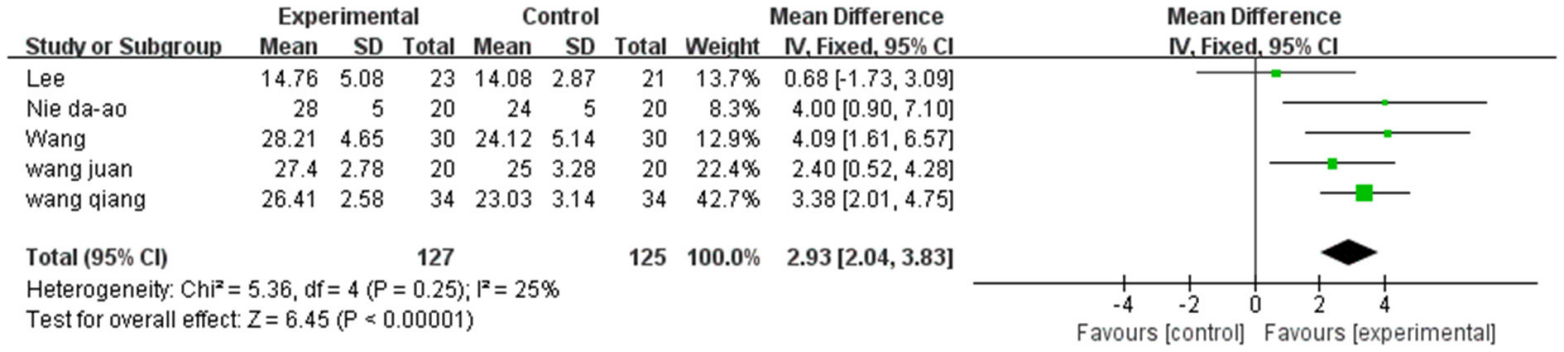
Forest plot of FMA for meta-analysis.

A total of 252 participants were included in five studies that used FMA. The results showed *P* = 0.26 and *I*^2^ = 25%, thus, we used a fixed-effect model [MD = 2.93, 95% CI (2.04, 3.83), (*P* < 0.01)]. Herein, the difference between the two groups was statistically significant (Figure 7).

#### Balance ability

Balance ability was described using two aspects: BBS and OBI.

**Figure 8:**
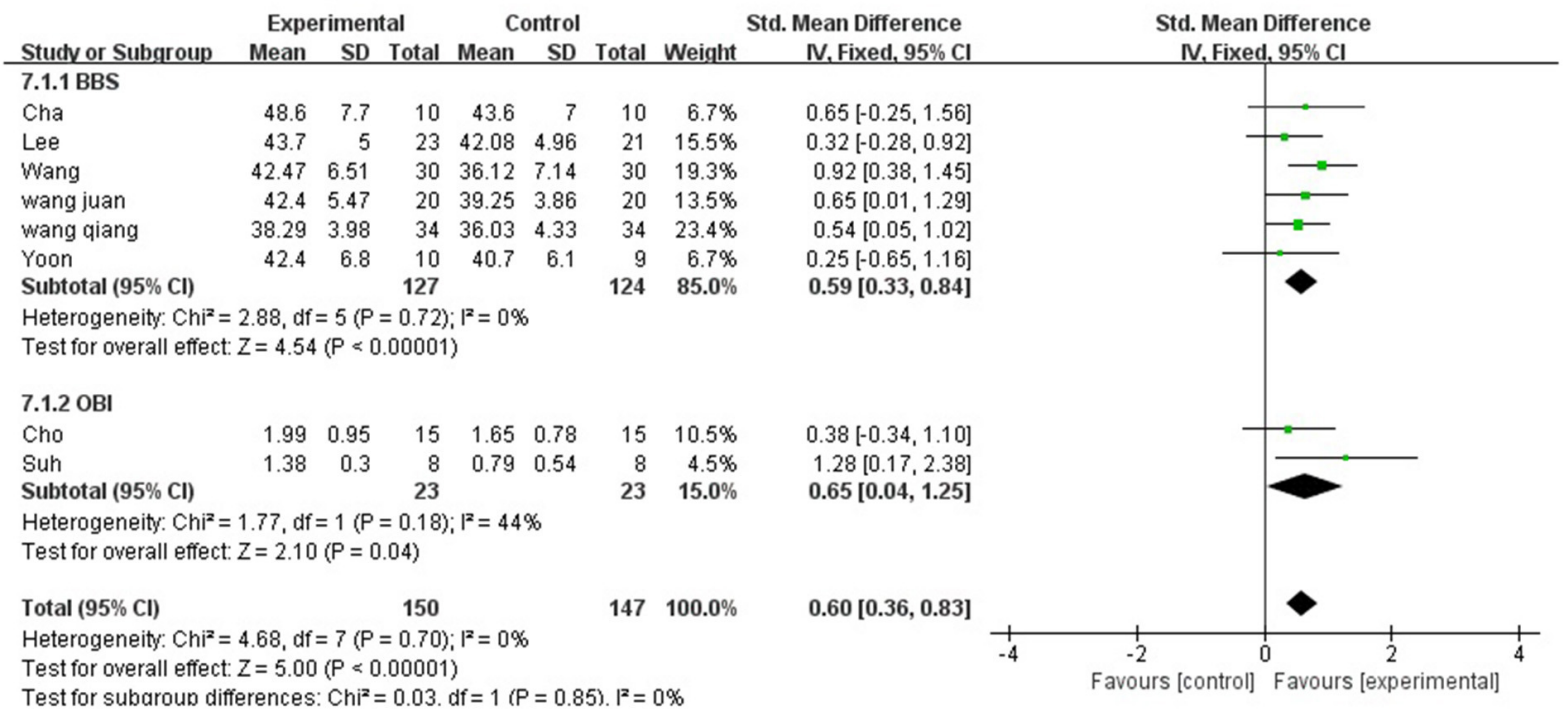
Forest plot of balance ability for meta-analysis.

A total of 297 participants were included in eight studies on balance ability. Considering that it is a combination of two indicators, hence, we selected the random-effect model [SMD = 0.60, 95% CI (0.36, 0.83), (*P* < 0.01)], heterogeneity *P* = 0.70 and *I*^2^ = 0%. We performed subgroup analysis as follows: based on the evaluation scale, the studies were divided into two subgroups: BBS and OBI. The subgroup of BBS was included in six studies. The results showed *P* = 0.72 and *I*^2^ = 0%, hence, we selected the fixed-effect model [MD = 0.59, 95% CI (0.33, 0.84), (*P* < 0.01)]. Meanwhile, the subgroup of OBI was included in two studies (6, 31). The results showed *P* = 0.18 and I^2^ = 44%, hence, we selected the fixed-effect model [MD = 0.65, 95% CI (0.104, 1.825), (*P* < 0.01)]. Therefore, the results of the subgroup analysis were statistically significant (Figure 8).

#### Funnel chart

**Figure 9:**
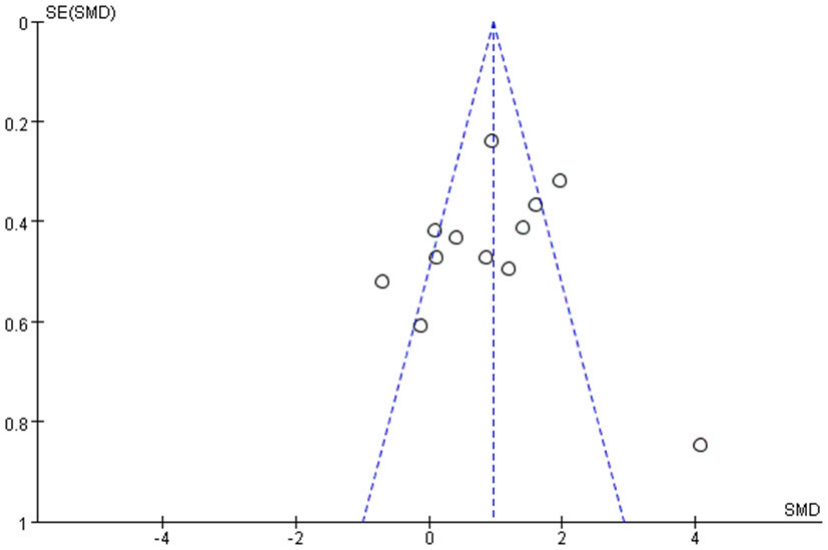
Funnel chart of step length for meta-analysis.

**Figure 10:**
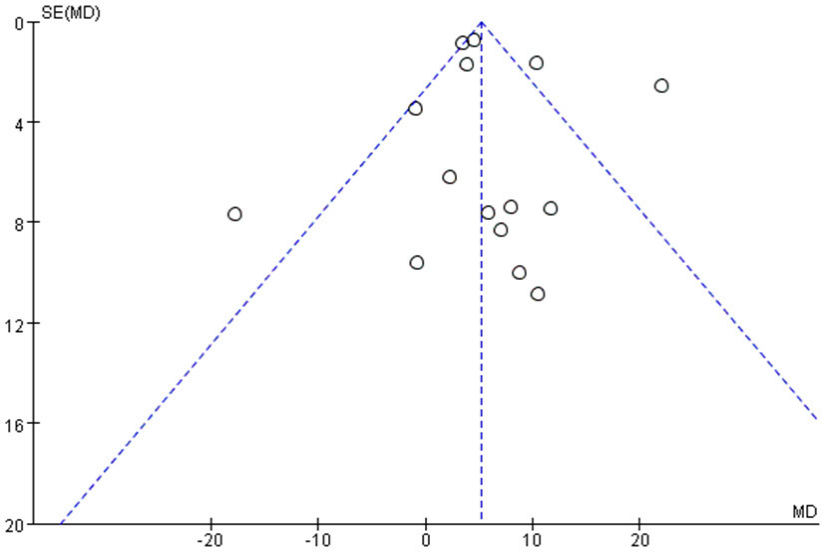
Funnel chart of step cadence for meta-analysis.

**Figure 11:**
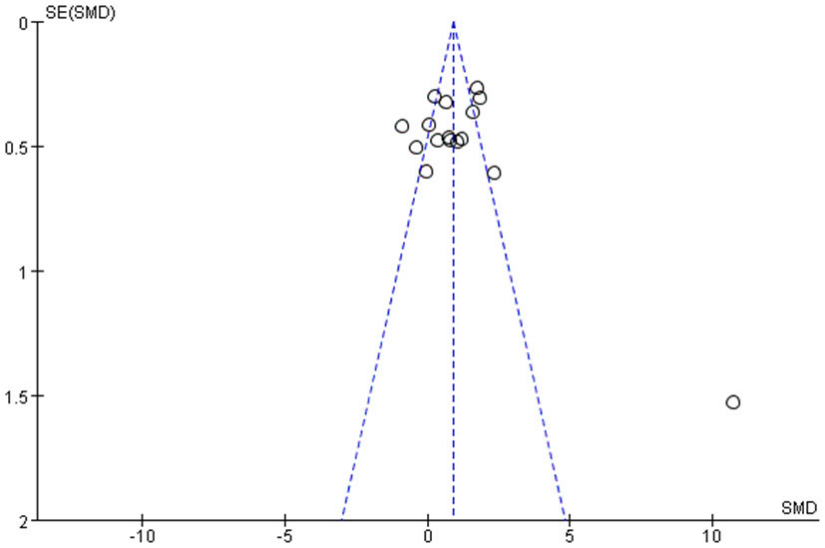
Funnel chart of velocity for meta-analysis.

Among all the outcome indicators, three indicators were included in more than 10 studies: step length (included 12 studies), step cadence (included 15 studies), and velocity (included 16 studies), hence, the funnel chart analysis was performed on these outcome indicators. The result of step length showed that four studies were outside the 95% interval, and the two sides of the funnel chart were asymmetrical (Figure 9). The result of step cadence showed that four studies were outside the 95% interval, and the two sides of the funnel chart were asymmetrical (Figure 10). Meanwhile, the result of velocity showed that eight studies were outside the 95% interval, and the two sides of the funnel chart were asymmetrical (Figure 11). Thus, these results all showed heterogeneity.

#### GRADE

The GRADEpro GDT online tool was used to evaluate the quality of evidence for the outcome indicators in the included studies. Herein, five outcome indicators were included, of which three and two were low- and moderate-quality indicators, respectively (Table 4).

**Table 4:** The quality of the evidence of outcome indicators.

**Certainty assessment**							**No. of patients**		**Effect**		**Certainty**	**Importance**
**No. of studies**	**Study** **design**	**Risk of** **bias**	**Inconsistency**	**Indirectness**	**Imprecision**	**Other consideration**	**RAS**	**Conventional treatment**	**Relative (95% CI)**	**Absolute (95% CI)**		
**Step length**												
12	Randomized trials	Not serious	Serious^a^	Not serious	Serious^b^	None	183	175	–	SMD 0.97 SD higher (0.74 higher to 1.2 higher)	⊕⊕○○ Low	
**Step cadence**												
15	Randomized trials	Not serious	Serious^a^	Not serious	Serious^b^	None	239	227	–	MD 5.16 higher (4.17 higher to 6.14 higher)	⊕⊕○○ Low	
**Velocity**												
16	Randomized trials	Not serious	Serious^a^	Not serious	Serious^b^	None	249	237	–	SMD 0.91 SD higher (0.71 higher to 1.11 higher)	⊕⊕○○ Low	
**FMA**
5	Randomized trials	Not serious	Not serious	Not serious	Serious^c^	None	127	125	–	MD 2.93 higher (2.04 higher to 3.83 higher)	⊕⊕⊕○ Moderate	
**Balance ability**												
8	Randomized trials	Not serious	Serious^a^	Not serious	Not serious	None	150	147	–	MD 0.26 lower (0.6 lower to 0.08 higher)	⊕⊕⊕○ Moderate	

CI, confidence interval; MD, mean difference; SMD, standardized mean difference.

^a^The confidence interval overlap is poor, the I2 value of the combined result is large, and the heterogeneity is moderate.

^b^The funnel graph is asymmetric, and there is a possibility of publication offset.

^c^The confidence interval is not narrow enough or there are few included studies.

## Discussion

This systematic review and meta-analysis aim to synthesize the current research progress and explore the effect of RAS on the gait, motor function, and balance ability of stroke patients. The current meta-analysis results show that RAS has a positive effect on improving gait, motor integration, and balance ability after stroke. The main research results are as follows:

(i) Under the intervention of rhythmic auditory stimulation, the gait parameters of patients were significantly improved and gait parameters (velocity, step length, and step cadence) were statistically significant (*P* < 0.01);
(ii) Under the intervention of rhythmic auditory stimulation, the motor function and balance ability of patients were significantly improved, and FMA, OBI, and BBS were also statistically significant (*P* < 0.01).
(iii) Among all the outcome indicators, three indicators were included in more than 10 studies: step length, step cadence, and velocity. The results showed that the two sides of the funnel chart were asymmetrical.

In terms of state parameters, the research results confirm the conclusions of the articles of Nascimento and Nascimento teams (Nascimento et al., 2015; Yoo and Kim, 2016), and RAS has certain efficacy in improving gait performance after stroke. From the perspective of neurophysiology, music can affect the nervous systems and motor function (Särkämö, 2018; Tramontano et al., 2021). Hence, music-related activities, such as listening to and making music, promote connections in brain regions that involve a large number of cortical and subcortical structures (Altenmüller and Schlaug, 2015; Tramontano et al., 2021). In particular, sound stimulation affects the limbic and paralimbic regions and the brain regions involved in motor function such as cortical motor areas (pre-motor and complement), cerebellum, and basal ganglia (Grahn, 2009; Lima et al., 2016). In addition, “entrainment” is another mechanism of action involved and is observed between sensory and motor systems in humans. Entrainment is defined as a temporal locking process in which one system's motion or signal frequency entrains the frequency of another system (Thaut et al., 2014). In the early 1990s, Thaut et al. established the role of entrainment in rehabilitation training and learning; wherein, the inherent periodicity of auditory rhythm patterns may affect motor patterns in patients with movement disorders (Thaut et al., 1999). Most importantly, an injured brain can enter into a rhythmic entrainment mechanism and rhythmic entrainment has been confirmed in clinical populations, such as gait training in hemiparetic stroke rehabilitation (Thaut et al., 1997) and Parkinson's disease (Thaut et al., 1996). RAS is a rehabilitation technique that involves using rhythmic cues (metronome or music with a rhythm) to promote rhythmic movements within the brain (Thaut et al., 1999). This technique typically uses a simple metronome beat that matches the patient's baseline gait but can also promote walking rhythm using a metronome beat embedded in a musical pattern that is 5–10% faster than the baseline (Thaut et al., 1996). Moreover, RAS can be used as an immediate entrainment stimulus that provides rhythmic cues during exercise. Rehabilitation programs use rhythmic auditory cues to enhance auditory-motor synchronization and promote sustained functional changes in movement (Schaffert et al., 2019).

In addition to the neurophysiological aspects of the system, RAS may have multiple effects on the musculoskeletal system (Ghai and Ghai, 2019). Meanwhile, Thaut et al. (2014) believe that the recruitment and activation of motor neurons are determined by the (central pattern generator, CPG) (Rossignol and Jones, 1976) of auditory neurons, which are affected by rhythm entrainment. Jun et al. (2014) also pointed out that stroke hemiplegic patients should start rhythmic walking training as early as possible to stimulate their “central pattern generator” (CPG). When specific sensory inputs are received, CPG generates the nerve rhythm impulses of alternate conversion between flexor and extensor during walking. First, the excitatory impulses of the flexor inhibit the extensor activity through the interneurons. After the completion of flexor excitation, the extensor nerve is excited and released, thereby causing the extensor activity, Thus, after the start of the walking action, the spontaneous alternating excitation of flexor extensor muscles will be generated and the walking action will also be generated.

Hence, this study also involves the analysis of the balance ability of stroke patients. The two indicators A and B observed in this study showed significant differences (*P* < 0.01), thereby suggesting that RAS may be effective in improving the balance ability of stroke patients. In the included study, there were few indicators or studies on balance ability. This may be because balance ability scales, such as BBS and OBI, require patients to have good motor functions to complete the test better. However, existing studies focus on early patients or patients with poor motor functions who cannot perform balance ability testing. This may be the reason why there are few results on balance in the included study.

The results of the meta-analysis showed that the heterogeneity of the three indicators (speed, stride length, and cadence) was very high in terms of gait parameters. Hence, heterogeneity cannot be reduced. The analysis believes that it may include the following aspects. First, the sample size of the included studies is generally low, and the sample size varies too much. For example, in the included studies, the maximum sample size is 78 people (Thaut et al., 2007), the minimum sample size is only 11 people (Yu-ge et al., 2016), and the small sample size is prone to false-positive results; in addition, the differences in intervention methods and intervention time may be the reasons for the high differences in this study. The types of intervention methods are different, including control and interventions between groups and treatment groups varied across studies [e.g., drug therapy, Neurodevelopmental Treatment (NDT), treadmill training, etc.] as well as in the duration of treatment interventions (time spans from 3 to 8 weeks) Finally, the degree of rigor of the experimental design of each study greatly varies. Herein, some studies are blinded, but some studies are not proposed, which may cause the high heterogeneity of the study.

## Future perspective

Stroke requires long-term or even life-long rehabilitation therapy (rehabilitation training). Therefore, changes in patients' daily living activities and home rehabilitation play an important role in their rehabilitation after a stroke. However, this study did not involve the observation of patients' daily living ability; in addition, the observation period was too short, thus, no long-term observation and follow-up report were provided. Therefore, whether or not the long-term efficacy of RAS is outstanding remains unclear. Based on the characteristics of disease recovery for stroke patients, home rehabilitation is very important. However, existing research and trial process is based on the medical care of medical staff in the hospital, which greatly avoids the occurrence of such events (such as falls), which is safe sexually guaranteed, whether safety at home can be guaranteed or whether RAS can be extended to home rehabilitation, and whether this clinical validity can be applied to home rehabilitation remains unclear. Therefore, further study should be conducted to assess the efficacy and safety of RAS in-home rehabilitation.

As an effective and safe home rehabilitation intervention, RAS can be used in home rehabilitation under the supervision and guidance of professional medical personnel if the safety and efficacy can be guaranteed. Through regular and professional periodic assessment, giving patients a rehabilitation policy for home rehabilitation using RAS cannot only save patients' time and energy but also reduce the economic burden on families and society.

## Study limitations

Our findings are based on articles written in English and Chinese. Articles in other languages were not included, which may have implications for our research. In addition, the research intervention factors are quite different, such as the intervention factors (type of RAS, combination method, etc.), intervention time, and so on, in some studies. Meanwhile, the intervention factors in some studies are the metronome combined with RAS, the rhythm of music, and so on, or even visual stimulation. Hence, these factors may affect the results of the study.

## Conclusions

This study suggests that RAS could improve the gait parameters, walking function, and balance function of individuals with stroke. However, studies or samples of outcome indicators for balance ability of stroke patients is relatively insufficient, which also requires further research in the future. Therefore, future studies should use a larger sample size and a more rigorous design to obtain strong conclusions about the advantages of RAS for the treatment of gait and motor function in stroke.

## Data availability statement

The original contributions presented in the study are included in the article/Supplementary material, further inquiries can be directed to the corresponding author.

## Author contributions

LW, J-lP, and A-lC: concept, idea, and research design. LW and J-lP: writing. LW, J-lP, and Y-jH: data collection. LW: data analysis. LW, J-lP, WX, Y-jH, and A-lC: consultation (including review of manuscript before submitting). All authors have read and approved the final manuscript.

## Conflict of interest

The authors declare that the research was conducted in the absence of any commercial or financial relationships that could be construed as a potential conflict of interest.

## Publisher's note

All claims expressed in this article are solely those of the authors and do not necessarily represent those of their affiliated organizations, or those of the publisher, the editors and the reviewers. Any product that may be evaluated in this article, or claim that may be made by its manufacturer, is not guaranteed or endorsed by the publisher.
